# Clinical benefit of adenosine as an adjunct to reperfusion in ST-elevation myocardial infarction patients: An updated meta-analysis of randomized controlled trials

**DOI:** 10.1016/j.ijcard.2015.09.005

**Published:** 2016-01-01

**Authors:** Heerajnarain Bulluck, Alex Sirker, Yoon K. Loke, David Garcia-Dorado, Derek J. Hausenloy

**Affiliations:** a The Hatter Cardiovascular Institute, Institute of Cardiovascular Science, NIHR University College London Hospitals Biomedical Research Centre, University College London, Chenies Mews, London, WC1E 6HX, UK; b The Heart Hospital, 16-18 Westmoreland Street, London W1G 8PH, UK; c University of East Anglia, Norwich Research Park, Norwich NR4 7TJ, UK; d Cardiology Department, Valld'Hebron Hospital, Universitat Autónomade Barcelona, Barcelona, Spain; e Cardiovascular and Metabolic Disorders Program, Duke-NUS Graduate Medical School, Singapore, Singapore; f National Heart Research Institute Singapore, National Heart Centre Singapore

**Keywords:** Primary percutaneous coronary intervention, Adenosine, ST-segment elevation myocardial infarction, Reperfusion injury, Adjunctive therapy

## Abstract

**Background:**

Adenosine administered as an adjunct to reperfusion can reduce coronary no-reflow and limit myocardial infarct (MI) size in ST-segment elevation myocardial infarction (STEMI) patients. Whether adjunctive adenosine therapy can improve clinical outcomes in reperfused STEMI patients is not clear and is investigated in this meta-analysis of 13 randomized controlled trials (RCTs).

**Methods:**

We performed an up-to-date search for all RCTs investigating adenosine as an adjunct to reperfusion in STEMI patients. We calculated pooled relative risks using a fixed-effect meta-analysis assessing the impact of adjunctive adenosine therapy on major clinical endpoint including all-cause mortality, non-fatal myocardial infarction, and heart failure. Surrogate markers of reperfusion were also analyzed.

**Results:**

13 RCTs (4273 STEMI patients) were identified and divided into 2 subgroups: intracoronary adenosine versus control (8 RCTs) and intravenous adenosine versus control (5 RCTs). In patients administered intracoronary adenosine, the incidence of heart failure was significantly lower (risk ratio [RR] 0.44 [95% CI 0.25–0.78], P = 0.005) and the incidence of coronary no-reflow was reduced (RR for TIMI flow<3 postreperfusion 0.68 [95% CI 0.47–0.99], P = 0.04). There was no difference in heart failure incidence in the intravenous adenosine group but most RCTs in this subgroup were from the thrombolysis era. There was no difference in non-fatal MI or all-cause mortality in both subgroups.

**Conclusion:**

We find evidence of improved clinical outcome in terms of less heart failure in STEMI patients administered intracoronary adenosine as an adjunct to reperfusion. This finding will need to be confirmed in a large adequately powered prospective RCT.

## 1 Introduction

Despite reperfusion by primary percutaneous coronary intervention (PPCI), the morbidity and mortality of ST-segment elevation myocardial infarction (STEMI) patients remain significant. This may be, in part, due to the presence of “myocardial reperfusion injury,” the term given to the tissue injury and cardiomyocyte death, which occurs on reperfusing previously ischemic myocardium and which contributes up to 50% of the final myocardial infarct (MI) size [1], [2]. Crucially, there is currently no effective therapy for preventing myocardial reperfusion injury, and as such novel therapies are required to target myocardial reperfusion injury so as to reduce MI size and preserve left ventricular systolic function thereby preventing the onset of heart failure.

Experimental studies have established that administering adenosine prior to index ischemia can reduce MI size in animal models of acute ischemia/reperfusion injury [3], but whether adenosine can also reduce MI size when administered at the time of reperfusion has been less clear [4], [5]. Although treatment with adenosine as an adjunct to reperfusion has been shown to prevent coronary no-reflow in STEMI patients, whether it can also limit MI size and improve clinical outcomes in this setting has been inconclusive [6], [7], [8], [9], [10], [11], [12], [13], [14], [15], [16], [17], [18]. Previous meta-analyses [19], [20], [21] have failed to find any benefit of adjunctive therapy with adenosine on clinical outcomes in STEMI patients. However, these meta-analyses did not include several recently published randomized control trials (RCT) [22], [16], [17], [18], including two studies reporting long-term clinical outcomes [23], [24]. Therefore, the aim of the current study was to perform an up-to-date meta-analysis of RCTs to determine whether adenosine administered as an adjunct to reperfusion improves clinical outcomes in STEMI patients.

## 2 Methods

This study was performed according to the recommendations specified in the Cochrane Handbook for Systematic Reviews of Interventions [25].

### 2.1 Eligibility criteria

All RCTs investigating the effect of adenosine (either intravenous or intracoronary) as an adjunct to reperfusion on clinical endpoints in STEMI patients were eligible for inclusion in the meta-analysis. RCTs comparing 3 arms were also included, provided we were able to assess data for the adenosine and control groups.

### 2.2 Search strategy

We searched MEDLINE and EMBASE databases up to November 2014. Additionally, we screened editorials and web-based sources of information to gain access to potential data from newly available or retrieved studies. The following search terms were used: “adenosine,” “adjunct,” “reperfusion injury,” “acute myocardial infarction,” “primary percutaneous intervention,” “randomized.” Attempt was made to contact authors of published RCTs when clinical endpoints were not reported.

### 2.3 Study selection

**Fig. 1:**
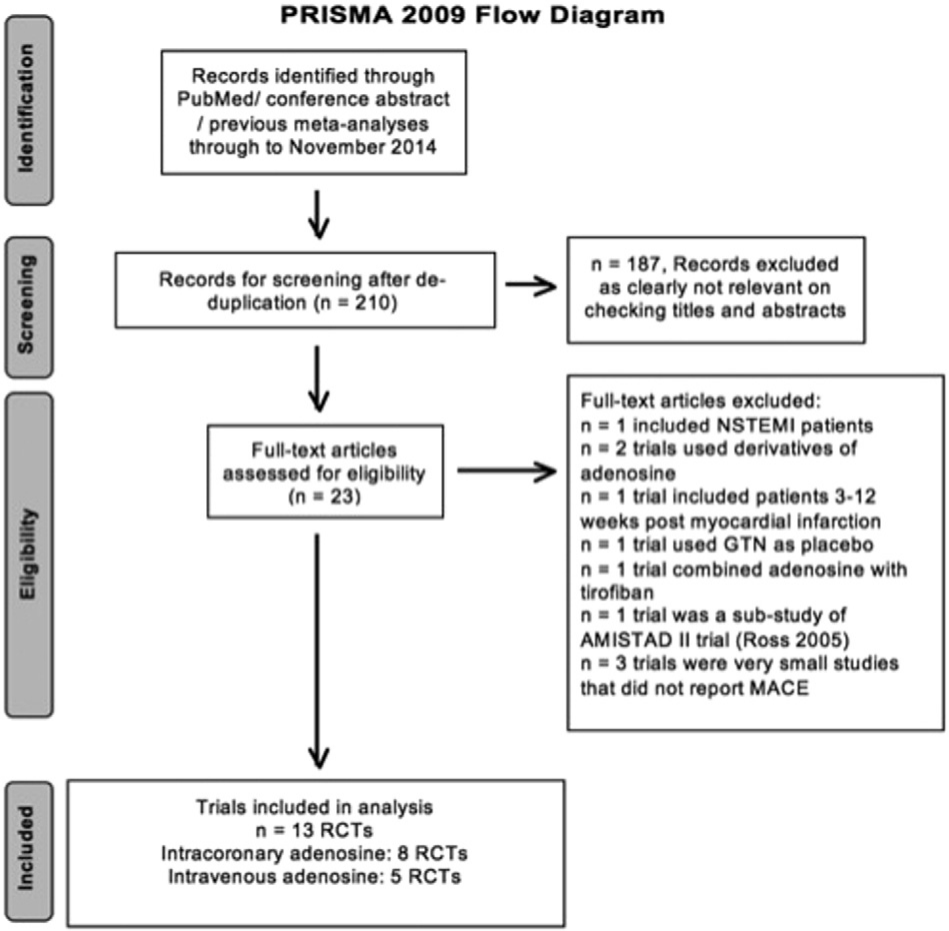
PRISMA 2009 flow diagram.

Two authors (HB, AS) identified suitable articles independently. Disagreement was resolved through consensus from a third investigator (DJH). Fig. 1 shows the process of study selection as per preferred reporting items for systematic reviews and meta-analyses (PRISMA) [26].

### 2.4 Data extraction and quality assessment

Baseline clinical characteristics of the study population, method of drug administration, and clinical outcome measures were extracted. Trial quality was determined as recommended by the Cochrane Handbook [25] (see Appendix A) but without constructing a composite quality score given the limitations inherent to such an approach [27]. We aimed to produce a funnel plot if there were > 10 included studies in the forest plots.

### 2.5 Endpoints and definitions

The main clinical endpoints analyzed were all-cause mortality, non-fatal myocardial infarction, and heart failure (defined as both heart failure during the initial hospitalization or rehospitalization for heart failure). Surrogate markers of reperfusion included ST-segment resolution, TIMI coronary flow < 3 postreperfusion, myocardial blush grade 0 or 1, and side effects of adenosine (second and third degree atrioventricular block and hypotension) were also analyzed.

### 2.6 Data synthesis and analysis

The RCTs were analyzed in 2 subgroups: intracoronary (IC) adenosine and intravenous (IV) adenosine. RevMan 5.2 (Nordic Cochrane Centre) was used to conduct a fixed-effect meta-analysis for the pooled risk ratio (RR), with 95% confidence intervals for dichotomous outcomes. We combined the different dose arms of adenosine in the pooled analysis against control. All reported P values are two-sided, with significance set at P < 0.05. Heterogeneity among trials was quantified using I^2^ statistics with I^2^ of 0–25%, 25–50% and 50–75% considered as low, moderate, and high heterogeneity, respectively.

### 2.7 Sensitivity analyses

If adenosine therapy showed a beneficial effect on a particular clinical endpoint, attempts were made to test the robustness of the result by removing one study at a time and looking at various subgroup analyses (trials using PPCI only; trials using thrombolysis only; trials performed after 2005 to account for changes and improvement in PPCI; excluding trials including patients presenting within 6 hours of symptoms onset only; excluding trials reporting outcomes during or after hospitalization only).

## 3 Results

### 3.1 Description of included studies

A total of 210 articles were retrieved from the search and 16 RCTs satisfied the predetermined inclusion criteria study review (Fig. 1). Of these, only 12 RCTs had investigated the effect of adenosine on clinical outcomes in STEMI patients. Furthermore, 1-year outcome data were obtained for the authors of the recently published PROMISE trial [22], which originally investigated the effect of intracoronary adenosine on infarct size by cardiac magnetic resonance. Two RCTs have subsequently reported clinical outcomes at 1 year [24], [23], and these were used for data extraction. Therefore, 8 RCTs using IC adenosine and 5 RCTs using IV adenosine were included in the meta-analysis. Table 1 shows the baseline characteristics of the 13 included RCTs. The study characteristics and the baseline demographics and inclusion and exclusion criteria are detailed in Table 1 and Appendix B, Appendix C.

Ji (2007) [28], Wang (2008) [29], and Akturk (2014) [30] were 3 very small trials with no clinical endpoints reported and were therefore not included in this analysis.

### 3.2 Quality assessment

The quality of the RCTs is shown in Appendix A. Randomization was assessed and considered adequate for 4 out of 13 trials. Although 8 of the studies were open-label, blinded observers independently adjudicated the endpoints in all of them. We did not formally test for publication bias, but we did attempt to directly contact investigators for clinical outcome data, which partly reduced the risk of publication bias.

### 3.3 Major clinical endpoints

**Fig. 2:**
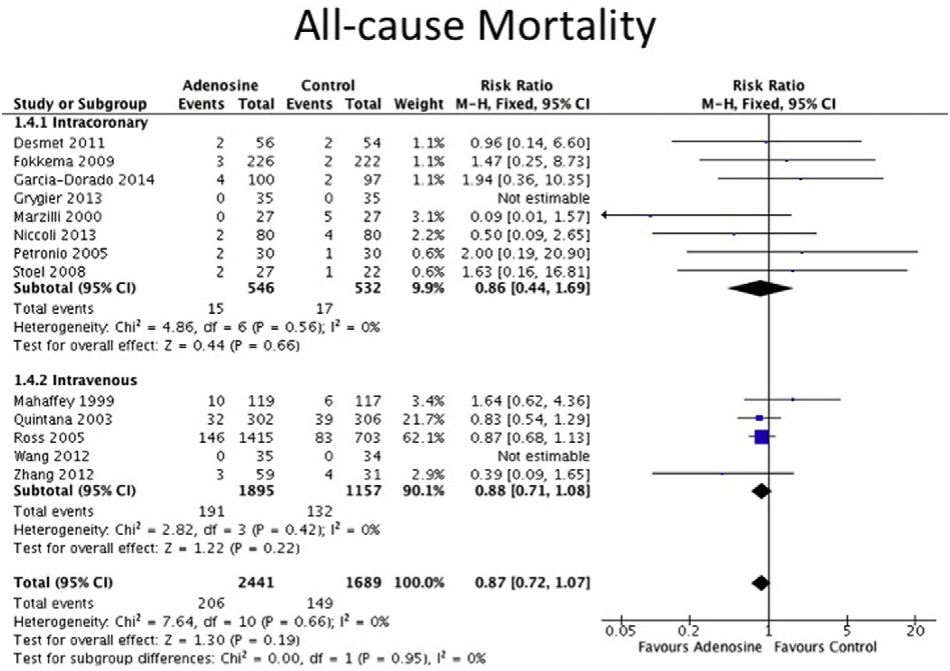
Forest plot for all-cause mortality, adenosine v control.

**Fig. 3:**
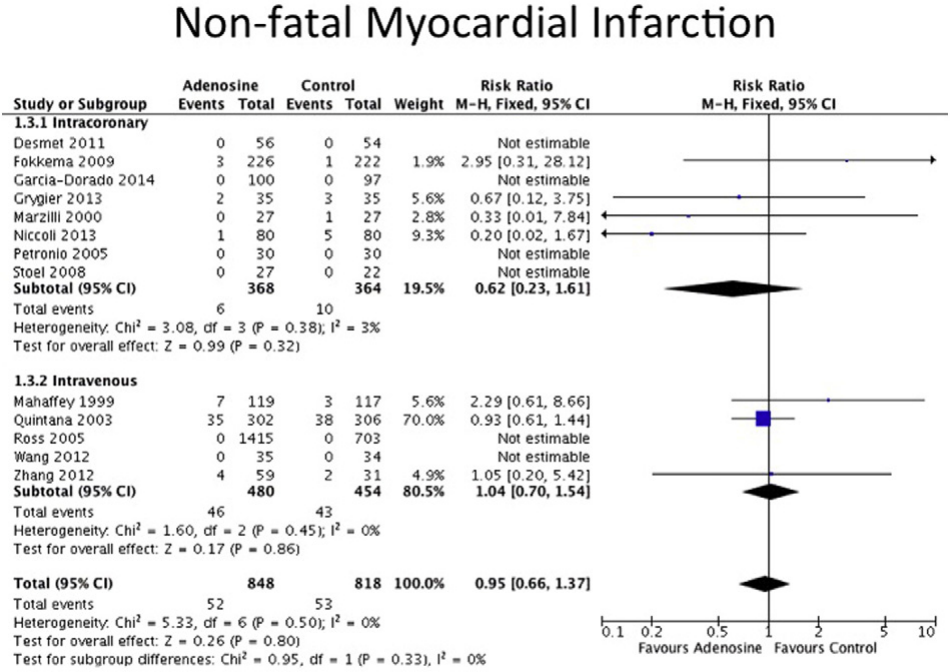
Forest plot for non-fatal MI, adenosine v control.

**Fig. 4:**
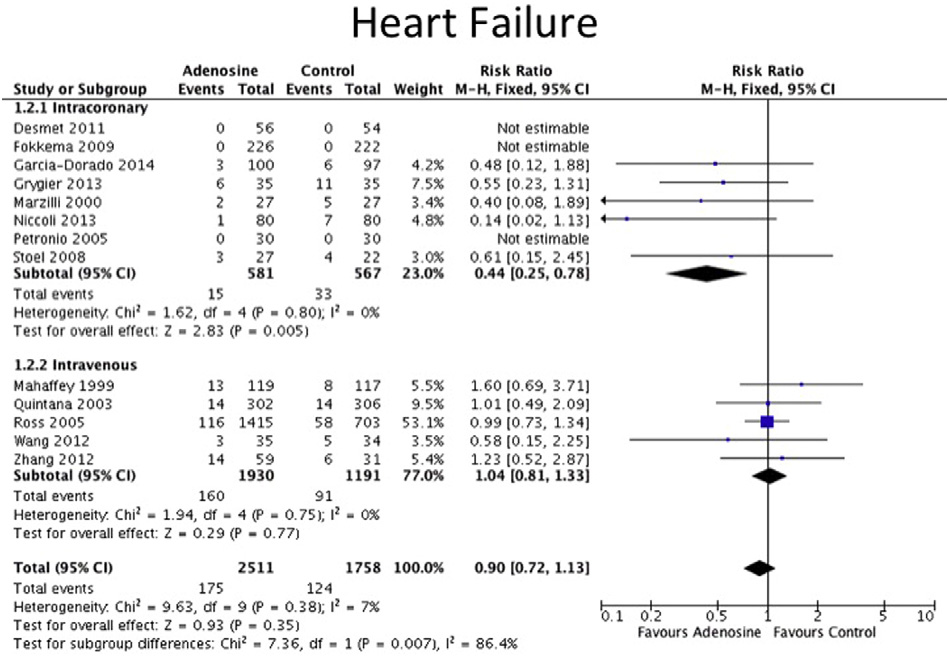
Forest plot for heart failure, adenosine v control.

The clinical endpoints are detailed in Table 2. All-cause mortality data were available for 7 out of 8 IC adenosine trials and for 4 out of 5 IV adenosine trials. Data on non-fatal MI were available for 4 out of 8 IC adenosine trials and for 3 out of 5 IV adenosine trials. There was no statistically difference in the incidence of non-fatal MI or all-cause mortality between adenosine and control for both routes of adenosine administration as shown in the Forest plots in Fig. 2, Fig. 3. The definitions for heart failure endpoints in each trial are listed in Table 3. Heart failure outcomes were available for 5 out of the 8 IC adenosine trials and for all of the IV adenosine trials as shown in Fig. 4. There was a reduction in heart failure outcomes in the IC adenosine subgroup (RR 0.44, 95% CI 0.25–0.78, P = 0.005) but no difference in the IV adenosine subgroup (RR 1.04, 95% CI 0.81–1.33, P = 0.36).

### 3.4 Surrogate markers of reperfusion and safety endpoints

**Fig. 5:**
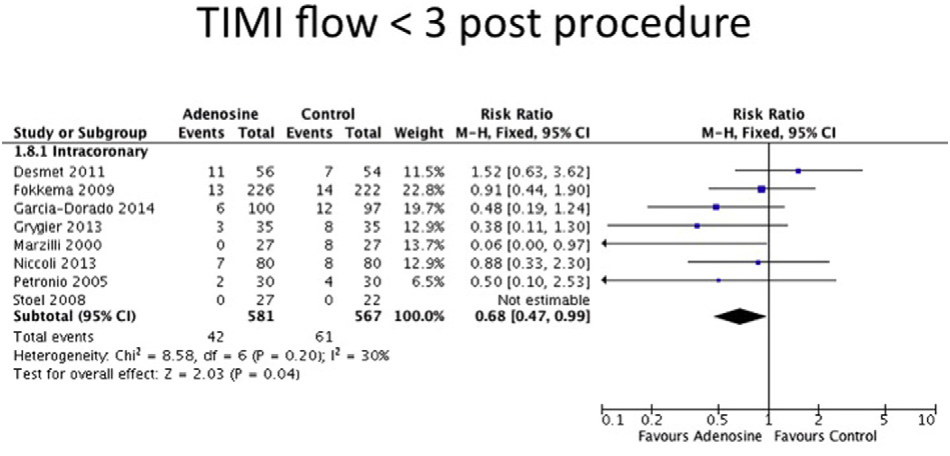
Forest plot for myocardial blush grade 0 or 1, adenosine v control.

**Fig. 6:**
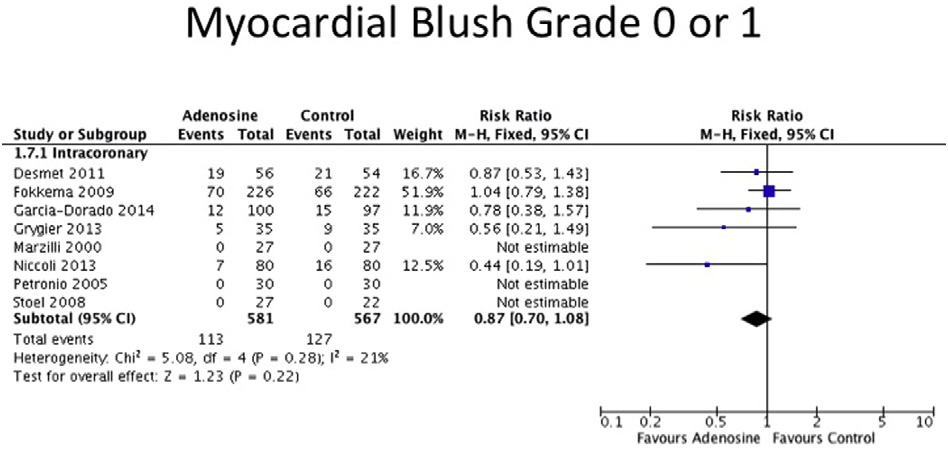
Forest plot for TIMI flow < 3, adenosine v control.

The details of the surrogate markers of reperfusion and safety endpoints for each trial are listed in Appendix D. Data on ST-segment resolution were available for 7 out of 8 IC adenosine trials only. However, as there was significant heterogeneity in the studies (Chi^2^ = 16.42, df = 6, P = 0.01; I^2^ = 63%), no summary effect size was estimated. Thrombolysis in myocardial infarction (TIMI) flow < 3 postprocedure was available in 7 out of 8 IC adenosine trials. TIMI flow < 3 postprocedure occurred with reduced incidence in the IC adenosine arm compared to control (RR 0.68 [95% CI 0.47–0.99], P = 0.04) (Fig. 5). Myocardial blush grade (MBG) of 0 or 1 was documented in 5 out of 8 IC adenosine RCTs. There was a trend toward less occurrence of MBG 0 or 1 in the adenosine group but this did not reach statistical significance (RR 0.87 [95% CI 0.70–1.08], P = 0.22) (Fig. 6). Examining these 5 studies in more detail, 400 μg of IC nitroglycerin was used in Fokkema et al. [13] in both arms prior to adenosine. Excluding this study from the analysis showed a lower incidence of MBG 0 or 1 in the adenosine group (RR 0.69, 95% CI 0.49–0.97, P = 0.03). As expected, both IV adenosine and IC adenosine were more likely to cause second and third degree heart block (IV adenosine: RR 2.86 [95% CI 1.63–5.02], P < 0.001; IC adenosine: RR 6.24, [95% CI 3.21–12.14], P < 0.001) and hypotension (IV adenosine: RR 1.19 [95% CI 1.03–1.38], P = 0.02; IC adenosine: not estimable) but these effects were transient in nature and none of the trials reported any long-lasting sequelae.

### 3.5 Sensitivity analyses

The reduction in the incidence of heart failure was still present in the IC adenosine subgroup despite removing one trial at a time; including trials using PPCI only (6 IC RCTs and 2 IV RCTs) and after only including trials published after 2005. This benefit persisted when only trials reporting outcomes after 6–12 months follow-up (5 IC RCTs) were considered. When trials including patients with up to 12 hours of symptoms duration were considered (6 RCTs), this benefit in heart failure reduction was no longer present but there was a trend toward less heart failure when IC adenosine trials (3 RCTs) only were considered (Appendix E).

## 4 Discussion

We show for the first time, improved clinical outcomes in STEMI patients administered adenosine as an adjunct to reperfusion. Our meta-analysis found that IC adenosine given at the time of PPCI reduced the incidence of heart failure in STEMI patients. This finding was associated with improved myocardial reperfusion as evidenced by a lower incidence of coronary no-flow post-PPCI, confirmed by less postreperfusion TIMI flow < 3 and less occurrence of MBG 0 or 1 (after excluding one study [13] using IC nitroglycerin in both arms prior to adenosine which itself has been shown to improve the microvascular dysfunction [31] and may have contributed to the neutral result in MBG 0 or 1 with adenosine in that study). The beneficial effects of adenosine were confined to those STEMI patients in whom adenosine was given via the IC route with no positive effects found with intravenously administered adenosine. However 3 out of 5 RCTs [6], [8], [11] administering IV adenosine were also confounded by the fact that they were performed in the thrombolysis era and therefore there is inadequate RCTs in this subgroup to allow us to draw any meaningful conclusion regarding IV adenosine in the PPCI setting.

In our meta-analysis, we found that IC adenosine therapy reduced the incidence of heart failure (during index admission or rehospitalization for heart failure), but there was no benefit in other major clinical endpoints of death, non-fatal MI, or revascularization. This benefit was still present despite excluding one RCT [7] in the intracoronary group looking at heart failure during hospitalization only (hospitalization for heart failure was available at 1 year for the remaining 4 RCTs [22], [23], [24], [12] – Table 3) and excluding the unpublished follow-up data from the PROMISE trial [22]. The beneficial effect of adenosine on heart failure most likely relates to the impact of adenosine therapy of preventing myocardial reperfusion injury and reducing MI size, although a favorable effect on ventricular remodeling cannot be ruled-out. Adenosine, via various adenosine receptor agonists, has been shown to reduce reperfusion injury and subsequent infarct size in animal models through the activation of the reperfusion injury salvage kinase pathway [32]. It is also known to be a potent vasodilator [33], to have anti-inflammatory properties [34] and has been implicated in the blockade of the neutrophil-mediated processes that promote microvascular obstruction [35]. Therefore, through these pleiotropic effects, adenosine can reduce infarct size and microvascular obstruction (MVO) and reduce the risk of adverse LV remodeling and heart failure.

The main strength of our study over previously published meta-analyses [19], [21], [20] is the inclusion of several recently published clinical outcomes studies [24], [23], [18], [22].

The REFLO-STEMI trial [36] (240 patients) looking at the effect of IC adenosine, sodium nitroprusside, and standard therapy on infarct size and MVO by cardiovascular MRI has completed recruitment and the results from this study would add to the current evidence on the role of IC adenosine in PPCI.

## 5 Limitations

There are several limitations to our meta-analysis. Firstly, the duration of symptoms varied among the RCTs, which may have diluted any beneficial effect observed with adenosine. Although we did attempt to explore trials including patients presenting within 6 hours of symptom onset, the majority of patients recruited within that time frame were confounded by also being treated by thrombolysis. Secondly, the dose of IV and IC adenosine differed greatly between studies (Table 1), and so it is difficult to ascertain the optimal IC dose of adenosine that had the most benefit. Thirdly, the timing of adenosine administration varied between studies ranging from initiating the IV infusion prior to reperfusion, and others administering the IC injection after the last balloon inflation. Finally, the RCT Stoel 2008 [12] only included patients with suboptimal ST-segment resolution and used a very high dose of IC adenosine. However, this was a small study and did not weigh significantly in the various analyses.

## 6 Conclusion

In summary, our meta-analysis shows for the first time that IC adenosine administered as an adjunct to reperfusion can improve clinical outcome as evidenced by a reduction in the incidence of heart failure in STEMI patients. The findings from this study are especially important for STEMI patients given the fact that despite recent reductions in mortality, the incidence of heart failure in this patient group is increasing. We hope that the findings from our meta-analysis will add to the positive evidence supporting the benefits of adenosine as an adjunct to reperfusion in STEMI patients and pave the way for large-scale prospective RCTs to confirm this beneficial effect of adenosine on major clinical outcomes.

## Disclosures

No conflict of interests ot relationship with industry exists.

## Funding

This work was supported by the British Heart Foundation (FS/10/039/28270), the RoseTrees Trust, and the National Institute for Health Research University College London Hospitals Biomedical Research Centre.

## Figures and Tables

**Table:** 

Study	Randomization sequence generation (was the method of generating the random sequence stated?)	Allocation concealment (following randomization, was allocation of intervention satisfactorily concealed, e.g. remote or centralized center, sealed opaque envelopes?)	Blinding of participants, personnel, and outcome (what type of blinding, and any specific detail on who was blinded?)	What percentage of patients was lost to follow-up?	Missing outcome data (were there any prespecified outcomes in the methods section that the authors said they would assess and report, but we were unable to extract the data for?)
Garcia-Dorado 2014	The randomization sequence was performed in permuted block sizes of 5 and 5.	NA	Double blinded	17 patients	NA
Niccoli 2013/ Oct 2013	Through an envelope opened by a trainee	NA	Open label Blind examination	2 patients	NA
Grygier 2011/ 2013	NA	NA	Blinded outcome adjudication	NA	NA
Desmet 2011	Computer-generated randomization	List kept in a sealed envelope hospital pharmacy	Double blinded	NA	Data on major adverse cardiac events not presented
Fokkema 2009	NA	NA	Open label trial with blinded evaluation of endpoints	NA	NA
Stoel 2008	NA	NA	Double blinded	1 (+ 1 cross-over) patients	NA
Petronio 2005	Randomization in a sequential alternating fashion	NA	Blinded evaluation of endpoints	NA	NA
Marzilli 2000	NA	NA	Blinded evaluation of angiograms	NA	NA
Zhang 2012	NA	NA	Blinded evaluation of cardiac echo and perfusion imaging	< 20%	NA
Wang 2012	NA	NA	Blinded evaluation of clinical and angiographic data	NA	NA
Ross 2005	NA	NA	Double blinded	NA	NA
Quintana 2003	NA	NA	Double blinded	2 patients	NA
Mahaffey 1999	NA	NA	Blinded evaluation of imaging studies	NA	NA

**Table:** 

Study	Number of patients in adenosine group	Number of patients in control group	Follow-up	Age (mean)	Male (%)	Smoker (%)	Diabetes mellitus (%)	Prior MI (%)	LAD infarct (%)	Proximal occlusion (%)	Pre-PCI TIMI 0/1 (%)
Garcia-Dorado 2014	100	97	6 months	59	86	52	15	NA	NA	NA	100
Niccoli 2013/ Oct 2013	80	80	1 year	64	76	58	23	22	46	50	86 (TIMI 0)
Grygier 2011/ 2013	35	35	1 year	65	62	50	23	10	19	NA	All patients TIMI 0-2 pre-PCI
Desmet 2011	56	54	1 year	61	82	49	10	1	41	NA	72
Fokkema 2009	226	222	1 month	62	75	58	10	8	40		61
Stoel 2008	27	22	1 year	67	66	36	11	NA	51	NA	NA
Petronio 2005	30	30	6 months	58	85	52	20	NA	58	NA	100
Marzilli 2000	27	27	During hospitalization	60	80	NA	NA	13	52	NA	NA
Zhang 2012	59	31	6 months	63	81	59	33	NA	47	NA	96.3% of patients TIMI 0-2 pre-PCI
Wang 2012	35	34	1 month	57	83	46	19	0	65	NA	NA
Ross 2005	1415	703	6 months	60	73	NA	16	13	100	NA	NA
Quintana 2003	302	306	12 months	65	76	32	9	23	NA	NA	NA
Mahaffey 1999	119	117	4–6 weeks	58	72	81	22	15	39	NA	NA

Abbreviations: MI: myocardial infarction; LAD: left anterior descending artery; PCI: percutaneous coronary intervention; TIMI: thrombolysis in myocardial infarction.

**Table:** 

Study	Inclusion criteria	Exclusion criteria
Garcia-Dorado 2014	Over 18 years of age with a diagnosis of STEMI on ECG and receiving PCI within 6 hours of symptom onset.	Previous myocardial infarction and TIMI flow grade 1 on initial angiography. Patients with potential contraindications for adenosine and contraindications for MRI examination or for gadolinium administration (renal function b30 mL/min/1.73 m^2^) or those with life expectancy of less than 6 months.
Niccoli 2013/ Oct 2013	Symptom onset < 12 hours before enrolment, ST-segment elevation of at least 2 mm in 2 or more contiguous leads, and thrombolysis in myocardial infarction (TIMI) flow grade 0/1 at baseline angiography.	Age < 18 years, previous STEMI in the same territory of current admission, cardiogenic shock, pregnancy, history of renal failure (serum creatinine > 3 mg/dl), contraindications to contrast agents or other study medications, paced rhythm, frequent ventricular ectopy, left bundle branch block, pre-excitation or other conditions or artifacts interfering with interpretation of the ST segment, culprit lesion located in a bypass graft, stent thrombosis, unidentified culprit lesion, and left main disease.
Grygier 2011/ 2013	6 hours of symptom onset, TIMI flow 0–2	TIMI flow 3, patients with chronic obstructive pulmonary disease or asthma and those who had received previous thrombolysis were excluded..
Desmet 2011	Cardiac sounding chest pain of at least 20 minutes x, a time from onset of symptoms of < 12 hours, and an ECG showing ST-segment elevation of .0.1 mV in two or more limb leads or .0.2 mV in two or more contiguous precordial leads, or presumed new left bundle- branch block.	Contraindication to heparin, low-molecular-weight heparin or clopidogrel, anticipated difficult vascular access, cardiogenic shock, inability to give informed consent, high-grade atrioventricular block, severe asthma, treatment with theophylline, glibenclamide, or dipyridamole, prior coronary artery surgery, and participation in any investigational drug or device study within the past 30 days.
Fokkema 2009	Symptoms of chest pain suggestive for myocardial ischemia for at least 30 minutes, a time from onset of symptoms of < 12 hours before hospital admission, and an ECG showing ST-segment elevation of > 0.1 mV in 2 or more leads.	The presence of cardiogenic shock, existence of a life-threatening disease with a life expectancy of < 6 months, receiving pharmacotherapy for chronic obstructive pulmonary disease, or no informed consent.
Stoel 2008	Following successful (defined as TIMI flow grade 2 or 3 without residual dissections or stenosis > 30% and no angiographic evidence of embolisation) PCI for acute myocardial infarction, patients with suboptimal reperfusion (< 70% STRes with persistent ST-elevation > 2 mV in at least one anterior lead and > 1 mV in a nonanterior lead) more than 10 minutes after last balloon inflation could be included.	Excluded were patients with hemodynamic instability, prior myocardial infarction or an ECG unsuitable for calculation of STRes (left bundle branch block, paced or severe disturbed rhythm). In addition, patients with a history of obstructive pulmonary disease were excluded because of potential side effects of adenosine.
Petronio 2005	Presentation > 6 hours from symptom onset; chest pain lasting 30 minutes and resistant to nitrates; 0.2 mV ST-segment elevation in 2 contiguous leads on a 12-lead electrocardiogram; TIMI flow 0–1 in the infarct-related artery at the diagnostic angiogram; absence of contraindications to abciximab and adenosine	Significant left main coronary disease; cardiogenic shock; AMI due to bypass graft occlusion; thrombolytic therapy before angioplastyWithdrawals or losses to follow-up: not reported
Marzilli 2000	Referred for PTCA within 3 hours from the onset of AMI; the culprit lesion was suitable for PPCI; presented with a TIMI flow from 0 to 2; informed consent	TIMI 3 flow and having spontaneous reperfusion; a history of bronchospasm and/or undergoing therapy with theophylline derivatives; had received thrombolytics in the emergency room
Zhang 2012	(1) typical chest pain presenting within 12 hours of onset, with ST-segment elevation in at least two contiguous leads of > 0.2 mV in precordial leads, > 0.1 mV in limb leads, or new left bundle branch block (LBBB), (2) they were candidates for primary PCI treatment, (3) > 18 years of age.	(1) thrombolytic treatment before PCI treatment, (2) previous myocardial infarction, (3) a history of coronary artery bypass graft (CABG) or PCI, (4) coronary angiography confirmed multi-vessel disease, (5) hypotension with systolic blood pressure < 90 mmHg or cardiogenic shock, or persistent bradycardia with heart beat < 55 beats/min, or advanced atrioventricular block without a pacemaker, (6) clinical evidence of significant reactive airway disease (e.g., asthma), or (7) advanced cancer, or end-stage disease.
Wang 2012	Patient who had received primary PCI within 12 hours of the onset of STEMI.	A history of myocardial infarction or coronary artery bypass grafting; cardiogenic shock; left ventricular ejection fraction < 40%; creatinine > 3 mg/dL; a history or clinical evidence of bronchospastic lung disease; second-degree or greater atrioventricular block without a functional pacemaker; atrial fibrillation; and allergy to adenosine.
Ross 2005	Age over 18 years, reperfusion therapy (fibrinolysis or percutaneous intervention) within 6 hours of onset of ischemic type pain (≥ 30 minutes), and electrocardiographic evidence of anterior STEMI. Electrocardiographic requirements were either ≥ 2 mm of ST-segment elevation in at least two contiguous precordial leads or (presumed) new left bundle branch block.	Initiation of reperfusion therapy before initiation of study drug. MI precipitated by a condition other than atherosclerotic CAD. Systolic blood pressure < 90 mmHg including cardiogenic shock and not responsive to intravenous fluids. Sustained bradycardia. Clinical evidence of significant reactive airway disease. Greater than first-degree atrioventricular block without functional pacemaker. Received dipyridamole within 24 hours of e. Coexistent condition associated with a limited life expectancy. Participation in another clinical research study.
Quintana 2003	Patients < 80 years of age with characteristic chest pain presenting within 12 hours of onset, with ST-segment elevation > 0.1 mV in at least two contiguous leads and being candidates for thrombolytic treatment were eligible for enrolment. Patients had to be on beta-blockers or planning to receive beta-blockers to be eligible.	Age < 18 years, pregnancy or lactation, second-degree or greater atrioventricular block without permanent pacemaker and current enrolment in other clinical trials.
Mahaffey 1999	Patients presenting within 6 hours of the onset of chest pain (consistent with ischemia, lasting at least 20 minutes, and not relieved by sublingual nitroglycerin) who had ST-segment elevation > 0.1 mV in two contiguous leads, in whom the clinical decision was made to treat with thrombolytic therapy, were eligible for enrolment.	< 18 years or > 79 years, women known or suspected to be pregnant, lactation, dipyridamole treatment within the past 24 hours, systolic blood pressure < 100 mmHg, cardiogenic shock, underlying condition in which hypotension may be poorly tolerated, history or clinical evidence of bronchospastic lung disease or prior bronchodilator therapy, second-degree or greater atrioventricular block without functional pacemaker, LBBB, sustained bradycardia, current enrolment in other investigational drug studies, and patients unlikely to be available for follow-up.

Abbreviations: STEMI: ST-elevation myocardial infarction; TIMI: thrombolysis in myocardial infarction; STRes: ST-segment resolution

**Table:** 

Study	Angiographic data						ST-segment resolution on ECG		Postadenosine					
Postprocedural TIMI flow < 3		Angiographic no reflow/ slow flow		MBG 0–1		Bradycardia		2nd degree/ 3rd degree heart block		Hypotension	
Adenosine	Placebo	Adenosine	Placebo	Adenosine	Placebo	Adenosine	Placebo	Adenosine	Placebo	Adenosine	Placebo	Adenosine	Placebo
Garcia-Dorado 2014	6	12	0	3	12	15	66	45	2	4	2	0	1	0
Niccoli 2013/ Oct 2013	7	8	NA	NA	7	16	57	41	1	0	11	2	5	7
Grygier 2011/ 2013	3	8	0	3	5	9	27	15	8	0	NA	NA	0	0
Desmet 2011	11	7	NA	NA	19	21	24	21	5	7	13	5	NA	NA
Fokkema 2009	13	14	2	1	70	66	154	147	34	5	31	2	24	2
Stoel 2008	NA	NA	NA	NA	NA	NA	9	2	NA	NA	NA	NA	NA	NA
Petronio 2005	2	4	4	5	NA	NA	13	16	NA	NA	5	0	NA	NA
Marzilli 2000	0	8	1	7	NA	NA	NA	NA	0	0	0	0	0	0
Zhang 2012	1	4	3	11	2	9	NA	NA	7	1	12	1	12	3
Wang 2012	4	5	NA	NA	NA	NA	NA	NA	NA	NA	0	0	NA	NA
Ross 2005	NA	NA	NA	NA	NA	NA	NA	NA	38	16	56	7	263	98
Quintana 2003	NA	NA	NA	NA	NA	NA	NA	NA	79	86	3	5	79	86
Mahaffey 1999	NA	NA	NA	NA	NA	NA	NA	NA	19	12	5	3	44	36

Abbreviations: MBG: myocardial blush grade; TIMI: thrombolysis in myocardial infarction.

**Table:** 

Outcome: heart failure	Studies	Participants	Effect estimate Risk ratio (M–H, fixed, 95% CI)
PPCI only	8	799	0.59 [0.39, 0.92]
Thrombolysis (IV adenosine before reperfusion)	3	2962	1.13 [0.79, 1.62]
Trials after 2005	7	745	0.62 [0.39, 0.97]
Duration of symptoms up to 12 hours	6	1086	0.80 [0.51, 1.25]
Duration of symptoms up to 12 hours (IC group only)	3	319	0.38 [0.14, 1.04]
6–12 months follow-up	8	3381	0.85 [0.67, 1.09]
6–12 months follow-up (IC group only)	5	586	0.46 [0.25, 0.84]

**Table 1:** Study characteristics.

Study and year	Clinical setting Country	N	Adenosine dose	Follow-up	Outcomes
*Intracoronary adenosine*					
Garcia-Dorado 2014	STEMI undergoing PPCI Spain	201	IC 4.5 mg over 2 minutes distal to the lesion immediately before thrombectomy and direct stenting	6 months	Primary outcome: infarct size measured as total myocardial necrotic mass as determined by late enhancement on CMR imaging performed between 2 and 7 days postreperfusion. Secondary outcomes: differences between groups in ejection fraction and ventricular volumes on the baseline CMR, in ejection fraction, infarct size, and ventricular volumes on the CMR performed at 6 months, and the difference between groups in creatine-kinase MB peak at the index episode. MACE at 1 year⁎.
Niccoli 2013/ Oct 2013	STEMI undergoing PPCI Italy	160	IC 120 μg as a fast bolus followed by 2 mg over 2 minutes following thrombus aspiration	1 year	Primary endpoint: the incidence of ST-segment resolution > 70% on surface ECG at 90 minutes after PCI Secondary endpoints: angiographic MVO incidence and MACE rate at 1 year
Grygier 2011/ 2013	STEMI undergoing PPCI Poland	70	IC 2 mg LCA, 1mg RCA, immediately after crossing the lesion and after first balloon inflation	1 year	Primary endpoints: (1) ST-segment elevation resolution 60 minutes after PCI, (2) MBG at the end of procedure, and (3) final TIMI flow grade and TIMI frame count at the end of procedure Secondary endpoints: (1) the composite endpoint of death, recurrent MI, heart failure and clinically driven TVR during 1-month follow-up, and (2) the composite endpoint of death, recurrent MI, heart failure, unplanned hospitalization for heart failure and clinically driven TVR at 1 year
Desmet 2011	STEMI undergoing PPCI Belgium	110	IC 4 mg bolus	1 year	Primary endpoint: myocardial salvage, defined as the percentage of the AAR, which was not necrotic on MRI on Days 2–3 Secondary endpoint: MVO at Days 2–3, expressed as a percentage of the AAR. Other secondary endpoints were TIMI flow grade, TIMI frame count and myocardial blush grade at the end of PCI, ST-segment resolution on the ECG after PCI, MACE in hospital and at 30 days and at 1 year, recovery of LV function as assessed using MRI at 4 months, evolution of cardiac markers in the first 24 hours
Fokkema 2009	STEMI undergoing PPCI Netherlands	448	IC 2 × 120 μg after thrombus aspiration and after stenting	1 month	Primary endpoint: the incidence of residual ST-segment deviation < 0.2 mV, 30–60 minutes after PCI Secondary endpoint: ST-segment elevation resolution, myocardial blush grade, TIMI flow on the angiogram after PCI, enzymatic infarct size, and clinical outcome at 30 days
Stoel 2008	STEMI undergoing PPCI Netherlands	49	IC 60 mg in 5–10 minutes after last balloon inflation	1 year	ST-segment resolution and ameliorates angiographic parameters of coronary reflow (TIMI frame count, MBG, coronary blood flow, coronary vascular resistance). Follow-up for 12 months for clinical outcome
Petronio 2005	STEMI undergoing PPCI Italy	60	IC 4 mg before first balloon inflation	6 months	Primary endpoint was the prevalence of 6-month LV remodeling Secondary endpoints were the following:(1) the prevalence of angiographic no-reflow; (2) the final corrected TIMI frame count, (3) the percentage change in LVEDV at the 6-month follow-up
Marzilli 2000	STEMI undergoing PPCI Italy	54	IC 4 mg in 1 minute after balloon inflation	During hospitalization	Primary endpoints: feasibility and safety of intracoronary adenosine administration in the setting of primary PTCA and its effect on coronary blood flow Secondary endpoints, indexes of myocardial damage, including LV regional function, Q-wave MI, recurrence of angina, non-fatal MI, heart failure, and cardiac death were evaluated during hospitalization

*Intravenous adenosine*					
Zhang 2012	STEMI undergoing PPCI China	90	IV 50 and 70 μg/kg/min after the guide wire crossed the lesion for 3 hours	6 months	Primary endpoint: left ventricular function, and infarct size Secondary endpoint: occurrence of cardiac and non-cardiac death, non-fatal myocardial infarction, and heart failure at 6 months
Wang 2012	STEMI undergoing PPCI China	69	IV 50 μg/kg/min for 3 hours, started prior to stent implantation	1 month	To investigate the effect of intravenous adenosine on myocardial perfusion and segmental contractile function when administered as an adjunct to PPCI Clinical outcomes were evaluated in terms of the occurrence of MACE at 1 month
Ross 2005	STEMI undergoing PPCI/ thrombolysis 13 countries	2118	IV 50 and 70 μg/kg/min for 3 hours to be started within 15 minutes either of the start of fibrinolysis or before coronary intervention	6 months	Primary endpoint: new CHF beginning > 24 hours after randomization, or the first rehospitalization for CHF, or death from any cause within 6 months. Infarct size was measured in a subset of 243 patients by SPECT Secondary endpoints: all-cause and cardiovascular mortality within 6 months and those specific to the infarct size sub-study
Quintana 2003	STEMI undergoing thrombolysis Sweden	608	IV 10 μg/kg/min started with thrombolysis and maintained for 6 hours	12 months	Primary endpoint; global and regional left ventricular systolic and diastolic function by echocardiography Secondary endpoint: all-cause and cardiovascular mortality, and non-fatal myocardial infarction during 12 months of follow-up
Mahaffey 1999	STEMI undergoing thrombolysis USA/ Canada/ Argentina	236	IV max 70 μg/kg/min for 3 hours before thrombolysis together with lignocaine	4–6 weeks	Primary endpoint: infarct size as determined by SPECT imaging at 6 ± 1 days Secondary endpoints: MSI and a composite of in-hospital clinical outcomes (death, re-infarction, shock, congestive heart failure, or stroke)

Abbreviations: STEMI: ST-elevation myocardial infarction; PPCI: percutaneous coronary intervention; IV: intravenous; IC: intracoronary; MVO: microvascular obstruction; LV left ventricle; LVEF: left ventricular ejection fraction; MRI: magnetic resonance imaging; TIMI: thrombolysis in myocardial infarction; MACE: major adverse cardiovascular event; MBG: myocardial blush grade; TVR: target vessel revascularisation; LVEDV: left ventricular end diastolic volume; PTCA: primary transcutaneous coronary angioplasty

⁎ Unpublished follow-up data obtained from Garcia-Dorado 2014

**Table 2:** Clinical endpoints.

Study	Major adverse cardiovascular event		Target vessel/ lesion revascularisation/ stent thrombosis		Deaths		Non-fatal myocardial infarction		Heart failure	
Adenosine	Control	Adenosine	Control	Adenosine	Control	Adenosine	Control	Adenosine	Control
Garcia-Dorado 2014	NA	NA	3⁎	9⁎	4⁎	2⁎	0⁎	0⁎	3⁎	6⁎
Niccoli 2013/ Oct 2013	10	23	6	7	2	4	1	5	1	7
Grygier 2011/ 2013	8	16	3	3	0	0	2	3	6	11
Desmet 2011	NA	NA	NA	NA	2	2	NA	NA	NA	NA
Fokkema 2009	14	10	8	7	3	2	3	1	NA	NA
Stoel 2008	6	5	1	0	2	1	NA	NA	3	4
Petronio 2005	NA	NA	NA	NA	2	1	NA	NA	NA	NA
Marzilli 2000	5	13	NA	NA	0	5	0	1	2	5
Zhang 2012	19	11	NA	NA	3	4	4	2	14	6
Wang 2012	3	5	NA	NA	0	0	0	0	3	5
Ross 2005	231	126	NA	NA	146	83	NA	NA	116	58
Quintana 2003	61	64	NA	NA	32	39	35	38	14	14
Mahaffey 1999	22	16	NA	NA	10	6	7	3	13	8

⁎ Unpublished follow-up data obtained from Garcia-Dorado 2014

**Table 3:** Heart failure time points.

Study	Heart failure
Garcia-Dorado 2013 abstract only	Hospitalization for heart failure at 1 year⁎
Niccoli 2013/ Oct 2013	Hospitalization for heart failure at 1 year
Grygier 2011/ 2013	Not clearly defined. Heart failure at 1 year
Stoel 2008	Not clearly defined. Heart failure at 1 year
Marzilli 2000	Heart failure during hospitalization
Zhang 2012	Heart failure during hospitalization
Wang 2012	Heart failure at 1 month
Ross 2005	Heart failure during hospitalization and rehospitalization for heart failure during 6 months
Quintana 2003	Heart failure during hospitalization
Mahaffey 1999	Heart failure during hospitalization

⁎ Unpublished follow-up data obtained from Garcia-Dorado 2014.

## References

[1] Yellon D.M., Hausenloy D.J. (2007). Myocardial reperfusion injury. N. Engl. J. Med..

[2] Frohlich G.M., Meier P., White S.K., Yellon D.M., Hausenloy D.J. (2013). Myocardial reperfusion injury: looking beyond primary PCI. Eur. Heart J..

[3] Liu G.S., Thornton J., Van Winkle D.M., Stanley A.W., Olsson R.A., Downey J.M. (1991). Protection against infarction afforded by preconditioning is mediated by A1 adenosine receptors in rabbit heart. Circulation.

[4] Todd J., Zhao Z.Q., Williams M.W., Sato H., Van Wylen D.G., Vinten-Johansen J. (1996). Intravascular adenosine at reperfusion reduces infarct size and neutrophil adherence. Ann. Thorac. Surg..

[5] Donato M., Gelpi R.J. (2003). Adenosine and cardioprotection during reperfusion—an overview. Mol. Cell. Biochem..

[6] Mahaffey K.W., Puma J.A., Barbagelata N.A., DiCarli M.F., Leesar M.A., Browne K.F. (1999). Adenosine as an adjunct to thrombolytic therapy for acute myocardial infarction: results of a multicenter, randomized, placebo-controlled trial: the Acute Myocardial Infarction STudy of ADenosine (AMISTAD) trial. J. Am. Coll. Cardiol..

[7] Marzilli M., Orsini E., Marraccini P., Testa R. (2000). Beneficial effects of intracoronary adenosine as an adjunct to primary angioplasty in acute myocardial infarction. Circulation.

[8] Quintana M., Hjemdahl P., Sollevi A., Kahan T., Edner M., Rehnqvist N. (2003). Left ventricular function and cardiovascular events following adjuvant therapy with adenosine in acute myocardial infarction treated with thrombolysis, results of the ATTenuation by Adenosine of Cardiac Complications (ATTACC) study. Eur. J. Clin. Pharmacol..

[9] Micari A., Belcik T.A., Balcells E.A., Powers E., Wei K., Kaul S. (2005). Improvement in microvascular reflow and reduction of infarct size with adenosine in patients undergoing primary coronary stenting. Am. J. Cardiol..

[10] Petronio A.S., De Carlo M., Ciabatti N., Amoroso G., Limbruno U., Palagi C. (2005). Left ventricular remodeling after primary coronary angioplasty in patients treated with abciximab or intracoronary adenosine. Am. Heart J..

[11] Ross A.M., Gibbons R.J., Stone G.W., Kloner R.A., Alexander R.W., Investigators A.-I. (2005). A randomized, double-blinded, placebo-controlled multicenter trial of adenosine as an adjunct to reperfusion in the treatment of acute myocardial infarction (AMISTAD-II). J. Am. Coll. Cardiol..

[12] Stoel M.G., Marques K.M., de Cock C.C., Bronzwaer J.G., von Birgelen C., Zijlstra F. (2008). High dose adenosine for suboptimal myocardial reperfusion after primary PCI: A randomized placebo-controlled pilot study. Catheter. Cardiovasc. Interv..

[13] Fokkema M.L., Vlaar P.J., Vogelzang M., Gu Y.L., Kampinga M.A., de Smet B.J. (2009). Effect of high-dose intracoronary adenosine administration during primary percutaneous coronary intervention in acute myocardial infarction: a randomized controlled trial. Catheter. Cardiovasc. Interv..

[14] Desmet W., Bogaert J., Dubois C., Sinnaeve P., Adriaenssens T., Pappas C. (2011). High-dose intracoronary adenosine for myocardial salvage in patients with acute ST-segment elevation myocardial infarction. Eur. Heart J..

[15] Grygier M., Araszkiewicz A., Lesiak M., Janus M., Kowal J., Skorupski W. (2011). New method of intracoronary adenosine injection to prevent microvascular reperfusion injury in patients with acute myocardial infarction undergoing percutaneous coronary intervention. Am. J. Cardiol..

[16] Wang J., Chen Y.D., Zhi G., Xu Y., Chen L., Liu H.B. (2012). Beneficial effect of adenosine on myocardial perfusion in patients treated with primary percutaneous coronary intervention for acute myocardial infarction. Clin. Exp. Pharmacol. Physiol..

[17] Zhang H., Tian N.L., Hu Z.Y., Wang F., Chen L., Zhang Y.J., Chen S.L. (2012). Three hours continuous injection of adenosine improved left ventricular function and infarct size in patients with ST-segment elevation myocardial infarction. Chin. Med. J..

[18] Niccoli G., Rigattieri S., De Vita M.R., Valgimigli M., Corvo P., Fabbiocchi F. (2013). Open-Label, randomized, placebo-controlled evaluation of intracoronary adenosine or nitroprusside after thrombus aspiration during primary percutaneous coronary intervention for the prevention of microvascular obstruction in acute myocardial infarction: The REOPEN-AMI Study (intracoronary nitroprusside versus adenosine in acute myocardial infarction). J. Am. Coll. Cardiol. Intv..

[19] Singh M., Shah T., Khosla K., Singh P., Molnar J., Khosla S. (2012). Safety and efficacy of intracoronary adenosine administration in patients with acute myocardial infarction undergoing primary percutaneous coronary intervention: a meta-analysis of randomized controlled trials. Ther. Adv. Cardiovasc. Dis..

[20] Aung Naing K., Li L., Su Q., Wu T. (2013). Adenosine and verapamil for no-reflow during primary percutaneous coronary intervention in people with acute myocardial infarction. Cochrane Database Syst. Rev..

[21] Navarese E.P., Buffon A., Andreotti F., Gurbel P.A., Kozinski M., Kubica A. (2012). Adenosine improves post-procedural coronary flow but not clinical outcomes in patients with acute coronary syndrome: a meta-analysis of randomized trials. Atherosclerosis.

[22] Garcia-Dorado D., Garcia-Del-Blanco B., Otaegui I., Rodriguez-Palomares J., Pineda V., Gimeno F. (2014). Intracoronary injection of adenosine before reperfusion in patients with ST-segment elevation myocardial infarction: a randomized controlled clinical trial. Int. J. Cardiol..

[23] Niccoli G., Spaziani C., Crea F., Investigators R.-A. (2014). Left ventricular remodeling and 1-year clinical follow-up of the REOPEN-AMI trial. J. Am. Coll. Cardiol..

[24] Grygier M., Araszkiewicz A., Lesiak M., Grajek S. (2013). Effect of new method of intracoronary adenosine injection during primary percutaneous coronary intervention on microvascular reperfusion injury - clinical outcome and 1-year follow-up. Cardiology.

[25] Higgins J.P., Altman D.G., Gotzsche P.C., Juni P., Moher D., Oxman A.D. (2011). The Cochrane Collaboration's tool for assessing risk of bias in randomised trials. BMJ.

[26] Moher D., Liberati A., Tetzlaff J., Altman D.G., Group P. (2009). Preferred reporting items for systematic reviews and meta-analyses: the PRISMA statement. BMJ.

[27] Juni P., Witschi A., Bloch R., Egger M. (1999). The hazards of scoring the quality of clinical trials for meta-analysis. JAMA.

[28] Ji Z.G., Han J.M., Liu G., Liu K. (2007). Effect of adenosine on ischemia-reperfusion injury during percutaneous coronary intervention. J. Clin. Rehabil. Tissue Eng. Res..

[29] Wang X., Ding Z.J., Chen J.Z. (2008). 在急诊经皮冠状动脉介入治疗中腺苷的应用研究。. J. Clin. Med. Pract..

[30] Akturk I.F., Yalcin A.A., Biyik I., Sarikamis C., Caglar N.T., Erturk M. (2014). Effects of verapamil and adenosine in an adjunct to tirofiban on resolution and prognosis of noreflow phenomenon in patients with acute myocardial infarction. Minerva Cardioangiol..

[31] Ito N., Nanto S., Doi Y., Kurozumi Y., Natsukawa T., Shibata H. (2013). Beneficial effects of intracoronary nicorandil on microvascular dysfunction after primary percutaneous coronary intervention: demonstration of its superiority to nitroglycerin in a cross-over study. Cardiovasc. Drugs Ther..

[32] Hausenloy D.J., Yellon D.M. (2007). Reperfusion injury salvage kinase signalling: taking a RISK for cardioprotection. Heart Fail. Rev..

[33] Berne R.M. (1980). The role of adenosine in the regulation of coronary blood flow. Circ. Res..

[34] Ernst P.B., Garrison J.C., Thompson L.F. (2010). Much ado about adenosine: adenosine synthesis and function in regulatory T cell biology. J. Immunol..

[35] Olafsson B., Forman M.B., Puett D.W., Pou A., Cates C.U., Friesinger G.C. (1987). Reduction of reperfusion injury in the canine preparation by intracoronary adenosine: importance of the endothelium and the no-reflow phenomenon. Circulation.

[36] Nazir S.A., Khan J.N., Mahmoud I.Z., Greenwood J.P., Blackman D.J., Kunadian V. (2014). The REFLO-STEMI trial comparing intracoronary adenosine, sodium nitroprusside and standard therapy for the attenuation of infarct size and microvascular obstruction during primary percutaneous coronary intervention: study protocol for a randomised controlled trial. Trials.

